# Perineural invasion in head and neck cancers - a review-

**Published:** 2014-06-25

**Authors:** A Frunza, D Slavescu, I Lascar

**Affiliations:** * Bucharest Emergency Hospital, Bucharest, Romania; ** Carol Davila University of Medicine and Pharmacy, Bucharest, Romania

**Keywords:** perineural invasion, perineural spread, head and neck cancer, image-guided radiotherapy

## Abstract

Abstract

Rationale: perineural invasion is an under-recognized way of metastatic spread via tumoral invasion of the nerves. It is encountered in malignancies located in the head and neck but also in cancers involving the pancreas, colon and rectum, prostate, biliary tract and stomach. For some tumors, it may be the only way of metastatic spread. It represents a marker for poor outcome, with increased risk for locoregional recurrence and reduced survival rates. The molecular mechanisms behind this process are not yet fully understood; research is done to identify new therapeutic targets in order to achieve disease control. Objective: to make a rigorous analysis of this phenomenon and to highlight the best therapeutic approach. Method and results: a review of the current literature in order to harmonize the international protocols to our local pathology. Discussions: the surgical intervention is decisive to defeat the malignant process but must be associated with modern therapeutic methods, such as the image-guided radiation therapy and immunotherapy.

## Background

In order to understand the perineural invasion (PNI), we have to remind ourselves the biologic structure of a nerve. There are three components of the nerve sheath: the epineurium, the perineurium and the endoneurium. The outer layer, the epineurium, presents a rich vascular network (vasa nervorum) on the outside of the loose areolar connective tissue including lymphatics and on the inside, a dense structure made of collagen and elastin fibers. For many years, it was believed that these lymphatic vessels represented the path for neoplastic spread but recent studies have shown that the lymphatics do not penetrate the inner, dense aspect of the epineurium.

 According to Akert, the perineurium is a structure made of multiple layers of endothelial cells having a basement membrane on both sides. This perineurium separates the different fascicles of the nerve. Between the endothelial cells, tight junctions that transform this layer into a highly selective border are placed [**1-3**]. 

 The endoneurium delineates the individual nerve fibers. There are endoneurial blood vessels bordered by cells joined together with tight junctions, with no transendothelial channels. These features along with the characteristics of the perineurium explain the blood-nerve barrier [**4**].

 Pathogenesis and implications

 In an article published by Batsakis in 1985, we found the perineural invasion described as tumoral invasion in, around, and through the nerves [**5**]. It is generally accepted that at least 33% of the nerve circumference has to be surrounded by tumor cells to consider perineural invasion; anything less then 33% is just tumor proximity [**6**]. Therefore, we accepted PNI as a pathologic term related to microscopic perineural or endoneural tumor. The PNI is impossible to detect radiologically. On the other side, the perineural spread (PNS) means that the tumor cells have left the original site and travel along a nerve. Recent studies have demonstrated that PNI/PNS is done by signaling interactions between tumor cells and nerves; there are several neurotrophic factors involved, such as the brain-derived neurotrophic factor (BDNF), the glial cell line-derived neurotrophic factor (GDNF), the nerve growth factor (NGF) [**7-8**]. NGF stimulates epithelial cancer cell growth; through binding to tropomyosin receptor kinase A (a high-affinity receptor) it leads to over-production of matrix metalloproteinase 2, a proinvasive mediator. BDNF stimulates tumor cell invasion; it was found to be overexpressed in adenoid cystic carcinoma. GDNF has a chemotactic effect on tumor cells and increases the production of metalloproteinases (2 and 9) [**9**]. All these neurotrophins were considered potential therapeutic targets by means of monoclonal antibodies. 

 In head and neck cancers, the tumoral spread is often done along major nerves, which serve as conduits for intracranial extension. It is most commonly found in squamous cell carcinoma, followed by adenoid cystic carcinoma, lymphoma, rhabdomyosarcoma and implies decreased survival and high recurrence rates. The most affected nerves are the trigeminal and the facial nerve because of their extensive network of fibers in the head and neck area. They also present interconnections between them that may serve for tumor cell migration. 

 The first junction is the sphenopalatine ganglion; where branches of the maxillary nerve (the second trigeminal division branch) meet the Vidian nerve (the latter is derived from the greater superficial petrosal nerve, a facial nerve branch).

 The chorda tympani nerve (another VII th nerve branch) joins the lingual nerve (a division of the mandibular nerve, V3).

 The third communication is between the auriculotemporal branch of the mandibular nerve and the facial nerve within the parotid gland [**10**].

 Along these routes, head and neck cancers leave the primary site and travel retrogradely to reemerge at deeper intracranial destinations. When the cavernous sinus is reached, other cranial nerves may be involved, especially the oculomotor system.

 The perineural invasion and spread may happen in both centripetal/centrifugal way [**11**].

 The tumor cell migration may happen from one division of the trigeminal nerve to another via the bony channels and fissures. The first (ophthalmic) and second (maxillary) divisions are in close proximity at the orbital apex. The orbit also communicates by means of the inferior orbital fissure with the masticator space, so a lesion may spread from either the ophthalmic or the maxillary nerve to the mandibular nerve. The pterygomaxillary fissure is another communication between the masticator space (which contains the mandibular nerve) and the pterygopalatine fossa (the maxillary nerve).

 The trigeminal nerve may be a metastatic route for any of the three branches. The cutaneous malignancies spread via the sensory branches (for example from the forehead by the supraorbital nerve). The maxillary nerve is affected by nasopharyngeal tumors. The squamous cell carcinoma of the maxillary sinus especially gains access to the pterygopalatine fossa through the superior alveolar nerve or by penetration of the posterior wall. The mandibular nerve may be invaded by tumors from the lower lip, floor of the mouth and chin but also by orbital tumors via the inferior orbital fissure.

 The facial nerve is most commonly affected by carcinomas of the external ear or tumors arising from the parotid gland (adenoid cystic carcinoma, acinic or mucoepidermoid carcinoma) [**12**].

 The perineural invasion is often asymptomatic. Patients may experience pain, paresthesia, numbness and formication. Sometimes, in advanced cases, complete denervation may induce muscle atrophy. The Vth and VIIth facial nerves being the most frequently affected, the clinical presentation may include weakness of the muscles of mastication or facial expression. Loss of masseter muscle bulk may be observed.

 The PNS may initially be misdiagnosed as trigeminal neuralgia or Bell’s palsy [**13**].

 For these reasons, we rely on CT scanning and MRI as complementary methods to assess the perineural spread. With the help of the CT scan we can better observe the bones, given that a major part of the trigeminal or facial nerves is contained within bony structures. MRI has a better accuracy in differentiating normal tissue from tumor. It is also useful in differentiating inflammatory changes from the neoplasm. It provides a better soft tissue contrast and reduced artifact from dental hardware. Direct evidence of PNI on MRI is provided by the detection of enlargement and enhancement along a nerve [**14**].

 Enhancement is thought to be caused by the disruption of the blood-nerve barrier with nerve injury, with subsequently vascular congestion of endoneural capillaries and the breakdown of the perineural barrier. After a cranial nerve exits the skull through a foramen it is usually surrounded by fat. The disappearance of this fat is a sign of tumor invasion. Given this information, we should think to specifically evaluate these foramina in head and neck malignancies (the foramen rotundum, foramen ovale, the stylomastoid foramen, the mandibular foramen, the palatine foramen) [**15**]. In case of muscle denervation, this presents as increased signal uptake on T2 weighted images; in advanced cases, the muscle is replaced by fatty tissue.

 There are though some conditions that mimic PNS on imaging. The enhancement of the nerves may be encountered in infections (mucormycosis, aspergillus), sinonasal sarcoidosis, meningiomas close to foramin,a which may extend through them [**16**]. The head and neck oncology team is faced with the dilemma of deciding without pathologic confirmation of tumor spread because most of the times the perineural spread occurs in areas from we cannot take easily a biopsy.

 Treatment guidelines

 Most head and neck oncologists consider that the PNI represents an indication for adjuvant radiotherapy. There is evidence that the prognostic is poor even with radiotherapy. In a retrospective trial of 801 patients who received postoperative radiotherapy the locoregional control rate was 69% at 5 years in cases with PNI compared with 78% in those without PNI [**17**].

 Other systemic treatment options were cisplatin associated with radiotherapy [**18**]; in March 2006, the FDA approved the use of cetuximab (Erbitux ®) for use in combination with radiotherapy for squamous cell carcinoma of the head and neck. Cetuximab is a monoclonal antibody which binds the EDGF receptors of the tumor cell membrane; this stops them from stimulating the cancer cells to divide and grow. It may also make the cancer cells more sensitive to the effects of chemotherapy and radiotherapy. Two studies have evaluated the benefits of cetuximab in patients with squamous cell carcinoma of the head and neck in both the locally advanced (Bonner trial) and the recurrent and/or metastatic (EXTREME trial) settings. Erbitux was granted approval by the European Commission in November 2008 for the treatment of recurrent and/or metastatic squamous cell carcinoma of the head and neck based on the results of the EXTREME study.

 The surgical management in cases with PNS imposes the resection of the involved nerve until we have clear surgical margins. Unfortunately, many times PNS involves the skull base; skull base surgery is possible in selected patients but requires subspecialty surgical expertise (sometimes temporal bone resection is necessary) [**19**].

 The radiotherapy for PNS was hindered by the inability to shape the radiation by using 2D techniques. Critical structures as the brainstem or the optic nerves may be in close proximity to tumor and subsequently at risk for radiation injury. Nowadays, the conformal 3D radiotherapy enables the delivery of tumoricidal dose while the nearby structures are below the accepted radiation tolerance limits [**20**].

## Conclusions

Perineural invasion / spread pose difficult challenges to the multidisciplinary head and neck cancer team. The surgical excision must always follow the path of the involved nerve until clear margins are obtained. The adjuvant therapy is mandatory; the intensity-modulated radiation therapy and the image-guided radiation therapy offer the advantage of reduced toxicity and therefore a better quality of life for these patients.

The use of monoclonal antibodies such as cetuximab offers more specificity to treatment and remains a target for further research.
